# Supplementary material for: Modeling intrinsic factors of inclusive engagement in citizen science: Insights from the participants’ survey analysis of CSI-COP
Source: PLoS One. 2023 Nov 28;18(11):e0294575. doi: 10.1371/journal.pone.0294575 (PMC10684079; doi:10.1371/journal.pone.0294575)
Supplement: S1 File — (DOCX) [file pone.0294575.s001.docx]

Participant No.

To include recruiting partner acronym e.g. CU1, CTU3, BIU9, TIL20, etc.

**INFORMED CONSENT FORM:**

# Citizen Scientists Investigating Cookies and App GDPR compliance (CSI-COP)

You are invited to take part in this research study for the purpose of investigating cookies in websites and apps.

Before you decide to take part, you must **read the accompanying Participant Information Sheet.**

Please do not hesitate to ask questions if anything is unclear or if you would like more information about any aspect of this research. It is important that you feel able to take the necessary time to decide whether or not you wish to take part.

If you are happy to participate, please confirm your consent by circling YES against each of the statements below and then signing and dating the form as a CSI-COP project participant.

| **1** | **I confirm that I have read and understood the Participant Information Sheet for the above study and have had the opportunity to ask questions** | **YES** | **NO** |
| --- | --- | --- | --- |
| **2** | **I understand my participation is voluntary and that I am free to withdraw my data, without giving a reason, by contacting the lead researcher and the Research Support Office at any time until the date specified in the Participant Information Sheet** | **YES** | **NO** |
| **3** | **I have noted down my participant number (top left of this Consent Form) which may be required by the lead researcher if I wish to withdraw from the study** | **YES** | **NO** |
| **4** | **I understand that all the information I provide will be held securely and treated confidentially** | **YES** | **NO** |
| **5** | **I am happy for the information I provide to be used (anonymously) in academic papers and other formal research outputs** | **YES** | **NO** |
| **6** | **I agree to take part in the above study** | **YES** | **NO** |

**Thank you for your participation in this study. Your help is very much appreciated.**

| **Participant’s Name** | **Date** | **Signature** |
| --- | --- | --- |
|  |  |  |
| **Researcher** | **Date** | **Signature** |
|  |  |  |
