# Supplementary material for: Modeling intrinsic factors of inclusive engagement in citizen science: Insights from the participants’ survey analysis of CSI-COP
Source: PLoS One. 2023 Nov 28;18(11):e0294575. doi: 10.1371/journal.pone.0294575 (PMC10684079; doi:10.1371/journal.pone.0294575)
Supplement: S2 File — (DOCX) [file pone.0294575.s002.docx]

**PARTICIPANT INFORMATION SHEET**

Dear Citizen Scientist

We invite you to take part in research investigating online privacy by exploring cookies in websites you visit, and in apps you use on your smart phone. The official title of this project is ‘**Citizen scientists investigating cookies and app GDPR compliance’ (CSI-COP)**. Dr. Huma Shah, Director of Science of the CSI-COP project is at Coventry University, the lead partner of this international research and innovation project. Before you decide to take part as a citizen scientist it is important that you understand why the research is being conducted and what it will involve. Please kindly take the time to read the following information carefully.

**What is the purpose of the study?**

The General Data Protection Regulation (GDPR) is an EU law that requires organisations to safeguard personal data and uphold the privacy rights of anyone in the EU territory. Citizen scientists can play a valuable role in ensuring privacy and providing a better understanding of what information is tracked online. The CSI-COP project aims at a co-investigation between the professional researchers and citizen scientists interested in human rights in the digital age. CSI-COP is funded under the EU Horizon2020 scheme:

<https://cordis.europa.eu/project/id/873169>

CSI-COP project will mobilise citizen scientists from across Europe and beyond to investigate the different types of trackers in cookies and smart phone apps. The project will offer free training material to informally instruct citizen scientists on ‘informed consent’ and protections accorded in the GDPR. Citizen scientists in CSI-COP will be engaged in producing a taxonomy of trackers with the CSI-COP partners. This will lead to the co-creation of an open-access knowledge resource, a repository of digital trackers that can be searched by parents, teachers and more.

**Why have I been chosen to take part?**

CSI-COP aims to recruit a wide cohort of citizen scientists through a variety of online platforms, groups and interests. You are invited to participate in this study because you are interested in finding out the purpose of different cookies in websites and in smart phone apps.

**What are the benefits of taking part?**

Through participation in this project you will become aware of your human rights in the digital age (GDPR). By sharing findings with us of cookies you find in the websites you visit and in the apps you use on your smart phone, you will be helping CSI-COP and Coventry University to better understand the extent of online tracking. You will also be involved in designing a free to access online knowledge-base of cookies.

**Are there any risks associated with taking part?**

This study has been reviewed and approved through Coventry University’s formal research ethics procedure. There are no significant risks associated with participation.

**Do I have to take part?**

No – it is entirely up to you. If you do decide to take part, please keep this Information Sheet and complete the Informed Consent Form to show that you understand your rights in relation to the research, and that you are happy to participate. Please note down your participant number (which is on the Consent Form) and provide this to the lead researcher if you seek to withdraw from the study at a later date.

You are free to withdraw your information from the project data set at any time until the data are destroyed ten years after the conclusion of the project (30 June 2032). Please note, you will have the opportunity to co-author publications, including scientific articles. Hence please note that your data may be used in the production of these formal research outputs (e.g. journal articles, conference papers, theses and reports) prior to this date. Please be advised to contact the university at your earliest convenience should you wish to withdraw from the study. To withdraw, please contact the lead researcher (contact details are provided below). Please also contact the Research Support Office [[research.eec@coventry.ac.uk](mailto:research.eec@coventry.ac.uk); telephone [44(0)24 7765 7688](tel:+44(0)24%207765%207688) so that your request can be dealt with promptly in the event of the lead researcher’s absence. You do not need to give a reason. A decision to withdraw, or not to take part, will not affect you in any way.

**What will happen if I decide to take part?**

You will be asked a few questions before you take part, these will be regarding your age-range; gender, experience-level with the Internet. The questions will be asked in a survey you will receive prior to participation in one of CSI-COP’s free work-shops or online informal education ‘human rights in the digital age course’. The survey can be completed online (information on how and where to access to be advised), or can be completed in the clean, health-safe environment CSI-COP will provide before the start of any face-to-face workshop once the risk from the corona virus pandemic risk is greatly reduced. The survey should take 5-10 minutes to complete.

**Data Protection and Confidentiality**

Your data will be processed in accordance with the General Data Protection Regulation 2016 (GDPR) and for citizen scientists in the UK, the Data Protection Act 2018. All information collected about you will be kept strictly confidential. No personal, sensitive data will be collected. Unless they are fully anonymised in our records, your data will be referred to by a unique participant number rather than by name. Your data will only be viewed by the researcher/research team. All electronic data will be stored on a password-protected computer file in Coventry University’s servers. Any paper records will scanned-in to a digital document then stored securely. The paper files will be destroyed securely after the scanning process. Your consent information will be kept separately from the data you provide about your investigations. This in order to minimise risk in the event of a data breach. The lead researcher will take responsibility for data destruction and all collected data will be destroyed on or before 30 June 2032 [10 years after conclusion of CSI-COP].

**International Data Transfers**

Your data will not be stored or processed in any CSI-COP partner’s venue outside the UK. [Please be aware for yourself, that countries outside of the European Economic Area may not offer the same level of data privacy protection as in the UK].

**Data Protection Rights**

Coventry University is a Data Controller for the information you provide. You have the right to access information held about you. Your right of access can be exercised in accordance with the General Data Protection Regulation (GDPR) and the Data Protection Act 2018. You also have other rights including rights of correction, erasure, objection, and data portability. For more details, including the right to lodge a complaint with the Information Commissioner’s Office (ICO), please visit [www.ico.org.uk](http://www.ico.org.uk). Questions, comments and requests about your personal data can also be sent to the University Data Protection Officer: [dpo@coventry.ac.uk](mailto:dpo@coventry.ac.uk)

**What will happen with the results of this study?**

The results of this study may be summarised in published articles, reports and presentations. Quotes or key findings will always be made anonymous in any formal outputs unless we have your prior and explicit written permission to attribute them to you by name.

**Making a Complaint**

If you are unhappy with any aspect of this research, please first contact CSI-COP’s Director of Science, [Dr. Huma Shah, email: ab7778@coventry.ac.uk]. If you still have concerns and wish to make a formal complaint, please write to Dr. Paul Griffiths [LINE MANAGER] at this email: [ac7972@coventry.ac.uk](mailto:ac7972@coventry.ac.uk) .

CSI-COP hope ‘human rights in the digital age’ will interest you to participate in this timely project.

Dr. Huma Shah

Assistant Professor, School of Computing, Electronics and Mathematics

Associate Member, Centre for Data Science research

Coventry University

Coventry CV1 5FB

Email: ab7778@coventry.ac.uk

In your letter please provide information about the research project, specify the name of the researcher and detail the nature of your complaint.
