# Supplementary material for: Modeling intrinsic factors of inclusive engagement in citizen science: Insights from the participants’ survey analysis of CSI-COP
Source: PLoS One. 2023 Nov 28;18(11):e0294575. doi: 10.1371/journal.pone.0294575 (PMC10684079; doi:10.1371/journal.pone.0294575)
Supplement: S3 File — (PDF) [file pone.0294575.s003.pdf]

Certificate of Ethical Approval

Applicant:

Huma Shah

Project Title:

Citizen Scientists Investigating Cookies and App GDPR compliance

This is to certify that the above named applicant has completed the Coventry University Ethical Approval process and their project has been confirmed and approved as Medium Risk

Date of approval:

02 September 2020

Project Reference Number:

P109200
