# Supplementary material for: Modeling intrinsic factors of inclusive engagement in citizen science: Insights from the participants’ survey analysis of CSI-COP
Source: PLoS One. 2023 Nov 28;18(11):e0294575. doi: 10.1371/journal.pone.0294575 (PMC10684079; doi:10.1371/journal.pone.0294575)
Supplement: Attachment — Submitted filename: Modeling intrinsicfactors.docx [file pone.0294575.s004.docx]

- the paper subject " Modeling intrinsicfactors of inclusive engagement in Citizen Science: Insights from the participants’ survey analysis of CSI-COP" is significant.
- The authors claim properly placed in the context of the previous literature. This paper presents a new inclusive citizen science engagement model based on quantitative analysis of surveys administered to 540 participants of the dedicated free informal education course ‘Your Right to Privacy Online’ (MOOC - a massive online open course) from eight countries in the EU funded project, CSI-COP (Citizen Scientists Investigating Cookies and App GDPR compliance).
- But the authors need to retreat the introduction and support this research in this context. What is the difference between "Introduction" and "The objectives of CSI-Cop", the authors may have merged this to "Introduction"?
- "Theoretical framework of the study" the ten principles of CS needs revision and clear enough as a framework for this study.
- the manuscript well organized and written clearly enough to be accessible to non-specialists.
